# Dyspnea (breathlessness) in amyotrophic lateral sclerosis/motor neuron disease: prevalence, progression, severity, and correlates

**DOI:** 10.1080/21678421.2024.2322545

**Published:** 2024-03-11

**Authors:** Carolyn A. Young, Amina Chaouch, Christopher J. Mcdermott, Ammar Al-Chalabi, Suresh K. Chhetri, Kevin Talbot, Timothy Harrower, Richard W. Orrell, Joe Annadale, C. Oliver Hanemann, Antonio Scalfari, Alan Tennant, Roger Mills, ON BEHALF OF THE TONIC-ALS STUDY GROUP

**Affiliations:** 1 Walton Centre NHS Foundation Trust, Liverpool, UK; 2 Institute of Systems, Molecular and Integrative Biology, University of Liverpool, Liverpool, UK; 3 Greater Manchester Centre for Clinical Neurosciences, Salford, UK; 4 Sheffield Institute for Translational Neuroscience, Sheffield, UK; 5 Department of Basic and Clinical Neuroscience, Maurice Wohl Clinical Neuroscience Institute, King’s College London, London, UK; 6 Department of Neurology, King’s College Hospital, London, UK; 7 Lancashire Teaching Hospital, Preston, UK; 8 Nuffield Department of Clinical Neurosciences, University of Oxford, Oxford, UK; 9 Royal Devon and Exeter Foundation Trust Hospital, Exeter, UK; 10 Royal Free London NHS Foundation Trust, London, UK; 11 Hywel Dda University Health Board, Carmarthen, UK; 12 Plymouth University Peninsula Schools of Medicine and Dentistry, Plymouth, UK; 13 Imperial College Healthcare NHS Trust, London, UK, and; 14 Leeds Institute of Rheumatic and Musculoskeletal Medicine, University of Leeds, Leeds, UK

**Keywords:** Dyspnea, breathlessness, Rasch, trajectories of outcome in neurological conditions-ALS, measure

## Abstract

**Objective:**

Dyspnea, or breathlessness, is an important symptom in amyotrophic lateral sclerosis/motor neuron disease (ALS/MND). We examined the measurement properties of the Dyspnea-12.

**Methods:**

Rasch analysis enabled conversion of raw Dyspnea-12 scores to interval level metric equivalents. Converted data were used to perform trajectory modeling; those following different trajectories were compared for demographic, clinical, symptom, and functioning characteristics. Logistic regression examined differences between distinct trajectories.

**Results:**

In 1022 people, at baseline, mean metric Dyspnea-12 was 7.6 (SD 9.3). 49.8% had dyspnea, severe in 12.6%. Trajectory analysis over 28 months revealed three breathlessness trajectories: group 1 reported none at baseline/follow-up (42.7%); group 2 significantly increased over time (9.4%); group 3 had a much higher level at baseline which rose over follow-up (47.9%). Group 3 had worse outcomes on all symptoms, functioning and quality of life; compared to group 1, their odds of: respiratory onset sixfold greater; King’s stage ≥3 2.9 greater; increased odds of being bothered by choking, head drop, fasciculations, and muscle cramps; fatigue and anxiety also elevated (*p* < .01).

**Conclusion:**

Dyspnea is a cardinal symptom in ALS/MND and can be quickly measured using the Dyspnea-12. Raw scores can easily be converted to interval level measurement, for valid change scores and trajectory modeling. Dyspnea trajectories reveal different patterns, showing that clinical services must provide monitoring which is customized to individual patient need. Almost half of this large population had worsening dyspnea, confirming the importance of respiratory monitoring and interventions being integrated into routine ALS care.

## Graphical Abstract

**Figure:**
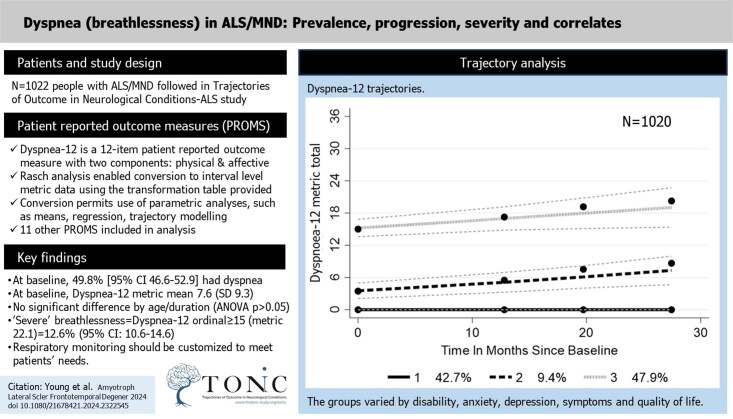

## Introduction

Dyspnea has been defined as “a subjective experience of breathing discomfort that consists of qualitatively distinct sensations that vary in intensity” (1). Dyspnea is common in amyotrophic lateral sclerosis/motor neuron disease (ALS/MND), particularly in the later stages. Patients use lay language such as breathlessness, rather than using the medical term dyspnea. A qualitative study found people with ALS (pwALS) believed that breathlessness indicated that the illness was a dangerous threat to their lives (2). In a recent study ranking functional domains, the most important domain reported by pwALS was respiratory (37.5%) (3). It has been argued that poor monitoring of respiratory function may lead to late initiation of noninvasive ventilation (NIV) in patients with ALS and that patient reported symptoms should be used for monitoring (4).

It has been pointed out that since dyspnea is a “perception of an abnormal or distressing internal state”, it can only be measured by patient self-report (5). There are many scales which measure or incorporate breathless-associated symptoms, some generic and some disease-specific. A systematic review of scales in chronic respiratory disease, cardiac disease, cancer, and ALS found that the majority of disease-specific scales were validated for chronic obstructive pulmonary disease, and few were applicable in other conditions (6). Since that time, the Amyotrophic Lateral Sclerosis Functional Rating Scale-Revised (ALSFRS-R) has provided a disease-specific respiratory domain, although several studies have raised issues about its validity (7,8). A disease-specific respiratory scale has been developed in the German language, the Dyspnea-ALS-Scale (DALS-15) (9). Another study introduced the ALS Respiratory Symptom Scale (ARES) (10). However, recently, two generic measures for dyspnea, the Multidimensional Dyspnea Profile (11) and the Dyspnea-12 (12), have been reported as standard instruments for measuring dyspnea in international trials (13). The Dyspnea-12 was derived from direct patient consultation and a systematic search of relevant literature on the language that patients use to describe dyspnea. Its items have been deemed to be relevant to the experience of dyspnea regardless of underlying disease and it is not activity dependent, rather measuring the direct impact that dyspnea has on a patient (14).

The Dyspnea-12 is included in the large-scale Trajectories of Outcomes in Neurological Conditions-ALS (TONiC-ALS) longitudinal study, providing data to investigate its reliability, validity, and invariance over time in those with ALS.

## Methods

### Samples

#### Main sample

Participants with ALS, diagnosed according to El Escorial World Federation of Neurology criteria for the diagnosis of ALS (15), were recruited into the TONiC-ALS study from specialist clinics across the United Kingdom from 2013, only data collected before 2020 was used in this analysis to avoid any influence from pandemic. Participants were excluded if they were unable to give informed consent or unable to complete the self-report questionnaire pack even with writing assistance from a scribe. Ethical approval was granted from the relevant local research committees (reference 11/NW/0743).

#### Calibration sample

A calibration sample was created using sequentially received questionnaires, split into “training” and “validation” samples. A sample size of approximately 500 in each was geared to maintaining a type I error rate of 5% for the Rasch fit statistics (16,17).

### Patient data

After an initial period to obtain ethical approvals for follow-up, the original questionnaires plus a change question, were sent out at intervals of at least 4 months. Onset type and duration from diagnosis were provided by clinical teams. In addition to demographic data, the pack included:

#### Patient reported outcome measures

- *Breathlessness:* The Dyspnea-12 is divided into seven “physical” and five “affective” items (12,18). Each item is scored 0–3, resulting in a total score of 0–36, where a high score represents extreme dyspnea.
- *Fatigue:* Neurological Fatigue Index-MND (NFI-MND) eight-item summary scale scored 0–24 with a high score represents greater fatigue (19).
- *Anxiety:* The Modified Anxiety Subscale of the Hospital Anxiety and Depression Scale (M-HADS-A), scored 0–18 where a higher score represents more anxiety, has modified cut points specific to ALS indicating “possible” and “probable” anxiety (20).
- *Functioning*: The ALSFRS-R is a measure of functioning, with 12 items asking about self-care, mobility, bulbar, and respiratory symptoms. With a range of 0–48, higher scores indicate less disability or better functioning (21).
- *Disability:* World Health Organization Disability Assessment Schedule (WHODAS 2.0) was used as the 32-item version omitting employment items, with a range of 0–128 (22).
- *Health status:* EQ-5D-5L utility value was derived from five items scored 1–5 with range −0.285 to 1, where higher scores indicate better health states (23,24).
- *Self-efficacy:* The General Self-Efficacy Scale (GSES) has 10 items each scored 1–4 where a higher score indicates higher self-efficacy (25).
- *Worry*: Penn State Worry Questionnaire has 16 items resulting in a range of 16–80, where a higher score indicates greater worry (26).
- *Social withdrawal:* MND Social Withdrawal Scale (MND-SWS) has 14 items with a range of 0–42, where higher scores indicate greater social withdrawal (27,28).
- *Stigma:* The Stigma Scale for Chronic Illness (SSCI-8) has score range 8–40 where a higher score indicates more experienced stigma (29).
- *Quality of life:* The World Health Organization Quality of Life-Bref (WHOQOL-Bref) has 24 items covering four domains: physical, psychological, social relationships, and environment. Two stand-alone questions on QOL and satisfaction with health are not included. A total score from the 24 items, obtained from a bi-factor solution, is used in this analysis (30).
- *Symptom inventory:* For the symptoms of fasciculations, muscle cramps, head drop, drooling, choking, and emotional lability, participants recorded whether they had the symptom and if so, whether it bothered them.

### Analytical procedures

#### Rasch analysis

Data from the Dyspnea-12 in the calibration sample were fit to the Rasch measurement model (31). A total score, as well as two subscales—physical and affective were investigated. A structured hierarchical analytical strategy was employed starting with simple item sets (level 1), through clusters of items (level 2), through to conceptual and other groupings (levels 3–6). These, along with all the relevant fit parameters, are described fully in the Supplementary File. Where data were shown to fit the Rasch model, the parameter estimates were imported into the full sample for further analysis.

#### Descriptive analysis

Correlates with various domains, as specified in the Wilson and Cleary model linking clinical variables to health-related quality of life were determined (32), together with the prevalence of breathlessness.

#### Trajectory analysis

A group-based trajectory model was assessed to ascertain if there are groups displaying different trajectories of breathlessness over time (33). Full details of the methodology are given in the Supplementary File.

#### Minimal important change (MIC) (also known as minimum clinically important difference (MCID)

Participants were asked to rate their level of breathlessness at baseline and first follow-up, as improved, stable, or worsened (34). The difference on the metric change score between worsening and improving was used to ascertain the MIC, expressed as median metric change of those who worsened (35).

## Results

### Full sample baseline

The full sample consisted of 1022 people giving 1636 records spread over up to eight time points. The mean age of participants at baseline was 64.9 years (SD 10.6), and 60.4% were male. Almost four-in-five (77.7%) were married. Just over one-quarter (27.2%) had bulbar onset, 70.6% limb onset, and 2.2% respiratory onset. Median duration was 9 months (IQR 3.7–22.9). Over half (55.8%) were at King’s stage 3 and above at baseline.

### Rasch analysis

Between the training and validation samples, there was no significant difference by age (*t*-test 0.489 (df 1020); *p* = 0.6244) or by gender, onset type or marital status (*χ*^2^, *p* > 0.05). However, there was a chance significant difference in duration with the training sample median at 11.7 months and the validation sample at 6.8 months (median test *χ*^2^ 22.4 (df 1); *p* ≤ 0.001).

**Figure 1.**
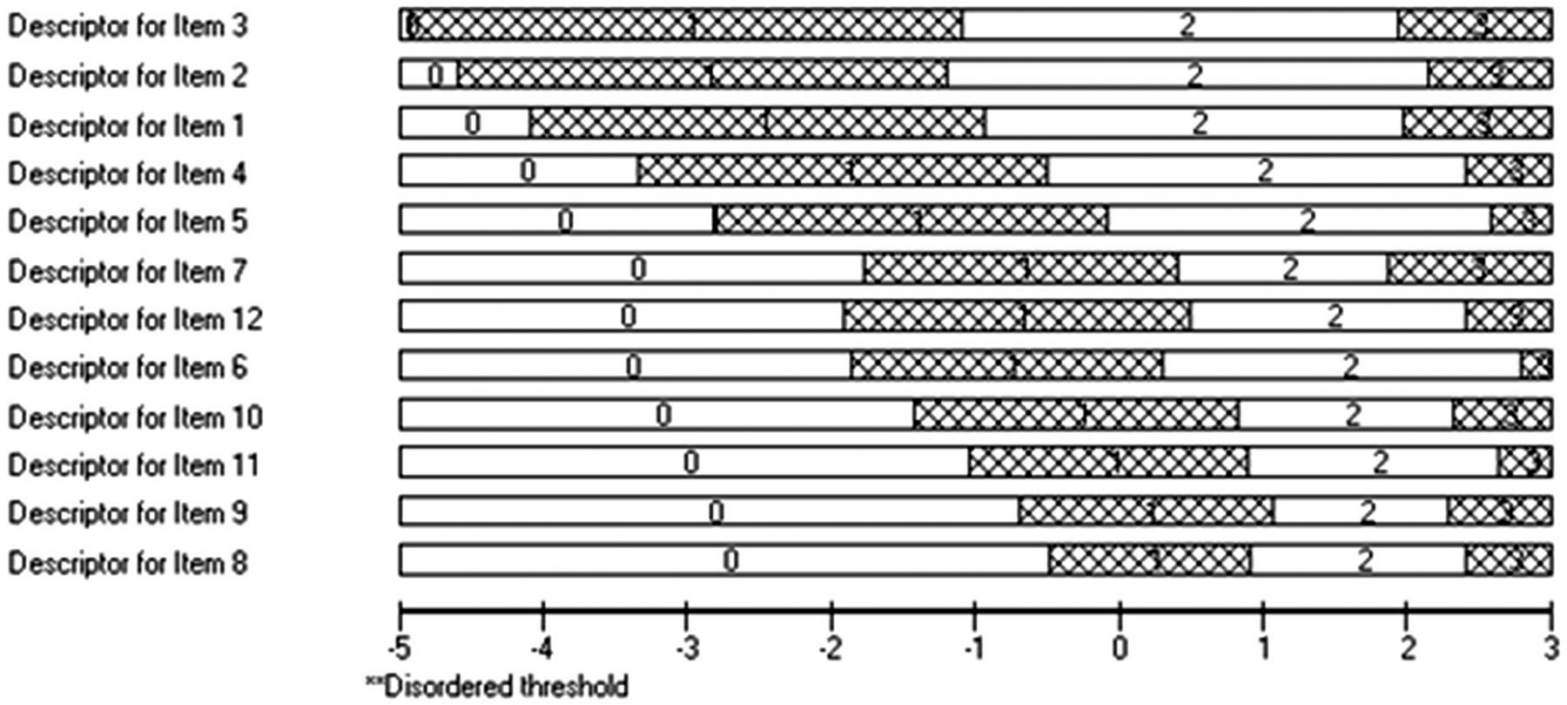
Dyspnea-12 item threshold map ordered by difficulty.

Fit of the data to the Rasch model is shown in Table 1. The 12 items in the training sample showed poor fit to the model with multi-dimensionality. Nevertheless, there was no DIF, and all item thresholds were fully ordered (Figure 1). Of note, the physical items (1–7) were the easiest to affirm, whereas the affective items (8–12) were much harder to affirm.

**Table 1.** Fit to the Rasch model.

Sample/domain	Fit residuals		Chi-square interaction fit			Reliability		Unidimensionality % *t* values (LCI)	DIF	ECV	Analysis level
	Items	Persons	Total	df	*p*	PSI	*α*				
Training total LD clusters	1.164	0.701	33.6	27	0.178	0.69	0.86	1.3	None	0.90	2
Validation total LD clusters	1.342	1.006	3.48	27	0.293	0.74	0.87	3.0	None	0.90	2
Total sample item set LD clusters	1.763	0.858	46.9	27	0.010	0.72	0.87	4.1	None	0.90	2
Training physical	0.884	0.987	91.6	63	0.011	0.85	0.95	4.0	None	–	1
Validation physical	1.965	1.165	79.3	63	0.080	0.87	0.96	5.1 (2.9)	None	–	1
Total physical sample LD clusters	2.225	0.829	47.6	27	0.009	0.79	0.91	3.2	None	–	2
Training affective	0.517	1.165	28.2	35	0.787	0.60	0.95	5.6 (2.9)	None	–	1
Validation affective LD clusters	0.736	1.275	42.1	28	0.034	0.68	0.92	4.0	None	0.96	2
Total affective sample	1.038	1.214	55.6	35	0.015	0.68	0.95	4.3	None	–	1

df: degrees of freedom; PSI: Person Separation Index; α: Cronbach’s alpha; LCI: Latent Correlation Index; DIF: differential item functioning; ECV: expected common variance; LD: local dependency.

*N* = 880.

Several clusters of locally dependent items were observed, for example, “My breath does not go in all the way” with “My breathing requires more work”. Consequently, four sets of locally dependent items were clustered into super items, and the data re-fit to the model, resulting in adequate fit at level 2 of the hierarchical analytical structure (Supplementary File). Here, a bi-factor equivalent solution was obtained which retained 90% of the explained common variance (ECV). This solution was replicated in the validation sample. The two samples were then merged to provide greater precision for the estimates, once again achieving satisfactory fit at level 2 analysis.

There was little difficulty fitting the physical subscale to the model in either the training or validation samples. When the samples were merged, the Chi-square fit was only marginal, but there were no other indications of mis-fit and so the solution was accepted at level 2. Likewise with the affective subscale, although due to the appearance of two locally dependent items in the validation sample, a level 2 cluster solution was required. The merged samples showed good fit at level 1.

A transformation table is provided to give the raw score-metric transformation for the total, physical, and affective scales (Table 2).

**Table 2.** Transformation table converting Dypnea-12 total raw score to interval level for total, physical, and affective scales.

Raw score	Dyspnea-12 Total	Dyspnea-12 Physical	Dyspnea-12 Affective
0	0.0	0.0	0.0
1	4.5	2.0	1.4
2	7.8	3.7	2.5
3	10.2	4.9	3.5
4	12.2	6.0	4.4
5	13.9	7.1	5.3
6	15.4	8.0	6.2
7	16.6	8.8	7.1
8	17.6	9.6	7.9
9	18.4	10.4	8.8
10	19.2	11.3	9.8
11	19.8	12.1	10.8
12	20.4	13.0	11.7
13	21.0	13.8	12.6
14	21.6	14.6	13.7
15	22.1	15.3	15.0
16	22.6	15.9	
17	23.0	16.6	
18	23.5	17.3	
19	23.9	18.1	
20	24.2	19.3	
21	24.5	21.0	
22	24.8		
23	25.1		
24	25.4		
25	25.6		
26	25.9		
27	26.1		
28	26.4		
29	26.7		
30	27.0		
31	27.4		
32	28.0		
33	28.7		
34	29.9		
35	32.1		
36	36.0		

Instructions for use of the transformation table. Providing the respondent has answered all the items, take the raw score and look across to the interval scale estimate for the relevant (sub)scale. For example, if you are converting the total Dyspnea-12, a raw score of 20 would give a standardized metric of 24.2. A raw Dyspnea-12 Physical of 20 gives a standardized metric of 19.3.

### Descriptive analysis

After importing the metric estimates into the full sample, the mean baseline Dyspnea-12 was 7.6 (SD 9.3) (Table 3). Almost half (49.8%) participants reported some level of breathlessness [95% CI 46.6–52.9]. There was no significant difference in the level of breathlessness by age group, nor by duration group (ANOVA, *p* > 0.05). There was significant difference by onset type, ranging from limb at 6.7, through bulbar at 9.2 to respiratory at 19.3 (ANOVA, *F* 25.9 (df2); *p* ≤ 0.001). Likewise, there was a significant difference between those at King’s stage 0–2 (3.8), and King’s stage ≥3+ (10.7) (*t*-test 12.6 (df1018); *p* ≤ 0.001). The Spearman correlation between Dyspnea-12 and ALSFRS-R respiratory domain was 0.77. Common comorbidities were hyperlipidemia (22.5%), hypertension (24.4%), and depression (11.7%). 9.4% reported a history of respiratory conditions (e.g. asthma, COPD), and the level of dyspnea differed significantly across those with or without respiratory comorbidity (*t*-test 5.8 (df 1016); *p* ≤ 0.001).

**Table 3.** Baseline trajectory characteristics.

Characteristic	Group 1	Group 2	Group 3	Total	Range
*Demographic*					
Age (years)	65.1	62.9	65.2	64.9*	16–90
% Female	41.5	28.1	40.2	39.6	0–100
% Married	76.3	84.4	77.6	77.7*	0–100
*Clinical*					
Duration (months)	25.5	19.6	19.0	21.8	0.3–295.7
* Subtype %*					
Bulbar	20.9	20.8	34.3	27.3	0–100
Limb	78.6	79.2	61.5	70.5	0–100
Respiratory	0.5	0.0	4.2	2.2	0–100
% King’s stage ≥3	38.5	45.8	73.1	55.8	0–100
*Symptoms*					
Breathlessness (Dyspnea-12)	0.0	3.0	15.4	7.6	0–36^a^
Fatigue (NFI-MND)	11.5	12.4	14.6	13.1	0–24^a^
* % bothered by*					
Fasciculation	16.6	21.1	20.8	19.0*	0–100
Muscle cramps	24.1	28.4	36.5	30.4	0–100
Head drop	6.8	9.5	23.1	14.9	0–100
Drooling	11.0	9.4	23.7	16.9	0–100
Choking	9.9	17.9	33.5	22.0	0–100
Emotional lability	14.4	20.8	22.0	18.6	0–100
*Functioning*					
ALSFRS-R	36.6	37.2	29.2	33.0	0–48
Bulbar	9.7	9.6	7.6	8.6	0–12
Limb	15.6	16.4	13.0	14.4	0–24
Respiratory	11.5	11.1	8.5	10.0	0–12
Disability (WHODAS 2.0)	51.9	49.9	64.8	57.9	0–128^a^
*Perceived health*					
Health status (EQ5D)	0.648	0.682	0.530	0.545	−0.27 to 1.0
*Quality of life*					
QOL (WHOQOL-Bref)	49.5	49.4	41.6	45.7	0–96
*Personal factors*					
Anxiety (M-HADS-A)	5.1	5.5	6.7	5.9	0–18^a^
Stigma (SSCI-8)	8.1	8.1	10.1	9.0	0–32^a^
Social withdrawal (MND SWS)	29.9	29.2	33.6	31.6	0–60^a^
Self-efficacy (GSES)	17.6	17.3	15.3	16.5	0–30
Worry (Penn State)	26.1	27.0	27.6	26.9*	0–64^a^
*N*	434	96	490	1020	

NFI-MND: Neurological Fatigue Index-MND; ALSFRS-R: Amyotrophic Lateral Sclerosis Functional Rating Scale-Revised; WHODAS: World Health Organization Disability Assessment Schedule; WHOQOL-Bref: World Health Organization Quality of Life-Bref; M-HADS-A: Modified Anxiety Subscale of Hospital Anxiety and Depression Scale; SSCI: Stigma Scale for Chronic Illness; MND SWS: MND Social Withdrawal Scale; GSES: General Self-Efficacy Scale.

*N* = 1020. All group comparisons are significant (Chi-square; ANOVA) except those marked with *. All PROMs measures except ALSFRS-R are metric.

^a^ Higher scores are worse.

### Follow-up sample

The first follow-up was completed by 331 people by 2019, mean age 64.9 years (SD 10.6). Taking part in follow-up did not vary by age (*t* = 1.075; (df 1016); *p* = 0.283), gender (*χ*^2^ 0.885 (df 1); *p* = 0.170), nor onset type (*χ*^2^ 2.838 (df 2); *p* = 0.242). However, those followed up had significantly lower baseline dyspnea (6.02) than those not (8.48) (*t* = 3.99 (df 1016); *p* ≤ 0.001). Worsening dyspnea was reported by 34.1% whereas 47.1% reported stability. The effect size for the change in Dyspnea-12 contrasting those who reported worsening compared to improvement was 1.3 (Glass’s delta), considered large. Of interest, while 9.6% had NIV at baseline, 23.4% did so at first follow-up (ALSFRS-R Q12). Between first and second follow-up, 38.6% reported worsening and 44.4% stable dyspnea. By the second follow-up, 12% of those without NIV at first follow-up had commenced it.

### Trajectory analysis

**Figure 2.**
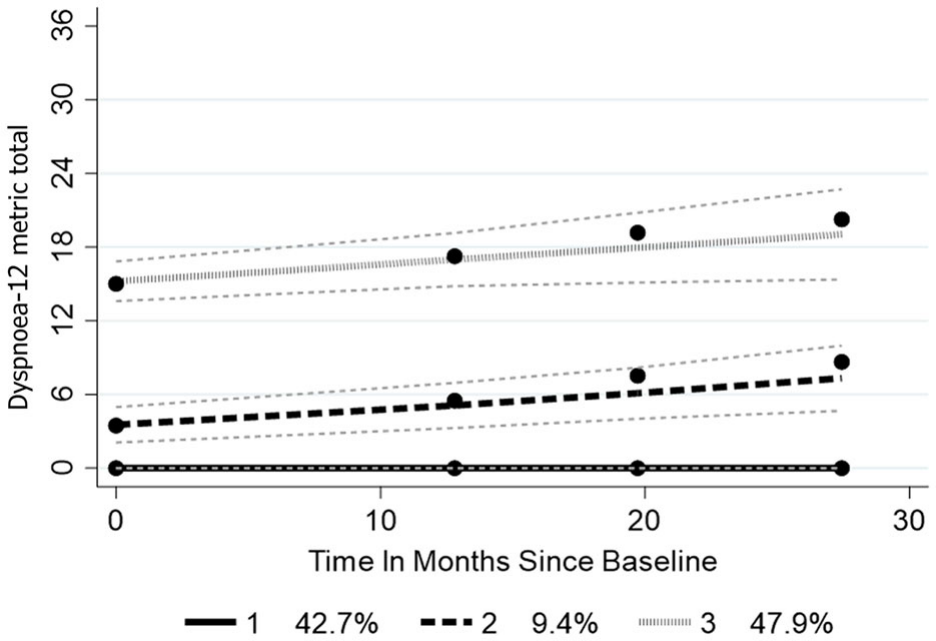
Dyspnea-12 trajectories. Fine dashed lines indicate 95% confidence limits.

Trajectory analysis over 28 months revealed three different trajectories consistent with the requirements laid out in the Supplementary File. Group 1 reported no breathlessness at baseline and during follow-up (Figure 2). While there is no significant difference between the baseline measurement (intercepts) of groups 1 and 2, group 2 had a significant increase over time. Group 3 entered the study with a much higher level of breathlessness which continued to rise over the follow-up. The slopes of increasing dyspnea of groups 2 and 3 are not significantly different (*t* = 0.329; df(585); *p* = 0.7419).

As group 3’s raw score upper quartile threshold was 15, for purposes of further analysis we define this upper quartile group as “severe” breathlessness with a Dyspnea-12 ordinal score of 15 and above (metric 22.1). This gives a prevalence of “severe” dyspnea at 12.6% (95% CI: 10.6–14.6). Note that 60.5% of this severe group is found at King’s stage 4b (i.e. with respiratory breathing support), and 86% at King’s stage 3 and above. As might be expected, group 3 contained the majority of those with respiratory onset, although respiratory onset is a small proportion of the group (Table 3). Although group 3’s duration at baseline was similar to group 2, but less than group 1, they displayed a significantly higher proportion of those at King’s stage 3 and above. Group 3 had severe, deteriorating dyspnea and displayed worse outcomes on all symptoms and functioning measures, as well as quality of life. Respiratory conditions were the only comorbidity which showed a significant difference across the three trajectory groups, largely confined to groups 2 and 3 (*χ*^2^ 24.1 (df 2); *p* < 0.001). Group 2 had a much longer engagement with the study than the other groups.

Given the baseline duration of group 1 was longer than that of group 3, and the levels of symptoms and functioning much better, a logistic regression looked at factors which may elucidate the magnitude of difference between the two groups (Table 4). There was no difference in any demographic factors between groups. The significant differences were that those in group 3 had over sixfold increase in odds of having respiratory onset compared to group 1; their odds of being at King’s stage ≥3 was increased by almost 2.9; they were more likely to have increased odds of being bothered by muscle cramps, head drop, and choking. Their odds of having fatigue and anxiety were also elevated, as was the odds of them being a past smoker. They were also less likely to report being religious than those in group 1 with a reduced odds of 0.598.

**Table 4.** Logistic regression for group 3 against group 1 as the reference.

	Odds ratio	Std. err	*t*-value	*p* Value	95% confidence interval		Sig.
Demographics							
Age	0.998	0.008	−0.20	0.839	0.982	1.015	
Gender: base, male							
Female	0.948	0.17	−0.30	0.766	0.667	1.347	
Marital status: base, married							
Other	1.031	0.207	0.15	0.878	0.696	1.529	
Clinical							
Onset type: base, bulbar							
Limb	0.561	0.137	−2.37	0.018	0.347	0.905	**
Respiratory	6.179	5.078	2.22	0.027	1.234	30.932	**
Duration	0.993	0.002	−3.07	0.002	0.988	0.997	***
King’s stage ≥3	2.855	0.533	5.62	0.000	1.98	4.118	***
Symptoms: *independent items, base, absent*							
* Fasciculation*							
Present, but not bothering	0.804	0.18	−0.98	0.328	0.518	1.246	
Bothering	0.526	0.165	−2.05	0.041	0.285	0.973	**
*Muscle cramps*							
Present, but not bothering	1.032	0.212	0.15	0.877	0.691	1.542	
Bothering	1.767	0.446	2.25	0.024	1.077	2.899	**
* Head drop*							
Present, but not bothering	1.551	0.484	1.41	0.159	0.842	2.859	
Bothering	2.017	0.541	2.61	0.009	1.192	3.414	***
* Drooling*							
Present, but not bothering	1.052	0.251	0.21	0.830	0.66	1.679	
Bothering	0.658	0.202	−1.37	0.172	0.36	1.2	
* Choking*							
Present, but not bothering	1.962	0.462	2.86	0.004	1.237	3.114	***
Bothering	4.005	1.111	5.00	0.000	2.326	6.899	***
* Emotional lability*							
Present, but not bothering	1.163	0.264	0.67	0.506	0.745	1.816	
Bothering	0.709	0.179	−1.36	0.173	0.432	1.162	
PROM-based symptoms							
Fatigue (NFI-MND)	1.078	0.021	3.77	0.000	1.037	1.12	***
Anxiety (M-HADS-A)	1.073	0.028	2.76	0.006	1.021	1.129	***
Personal							
Smoker: base, never							
Past smoker	1.53	0.318	2.04	0.041	1.018	2.301	**
Current smoker	1.22	0.502	0.48	0.628	0.545	2.733	
Unknown	1.007	0.206	0.04	0.971	0.675	1.503	
Religious: base, no							
Yes	0.598	0.102	−3.01	0.003	0.427	0.836	***
Constant	0.233	0.158	−2.15	0.031	0.062	0.878	**

*N* = 1015.

Std. err: standard error; PROM: patient reported outcome measure; NFI-MND: Neurological Fatigue Index-MND; M-HADS-A: Modified Anxiety Subscale of Hospital Anxiety and Depression Scale; McKelvey & Zavoina’s R2: 0.392.

*p < 0.1.

**p < 0.05.

***p < 0.01.

**Figure 3.**
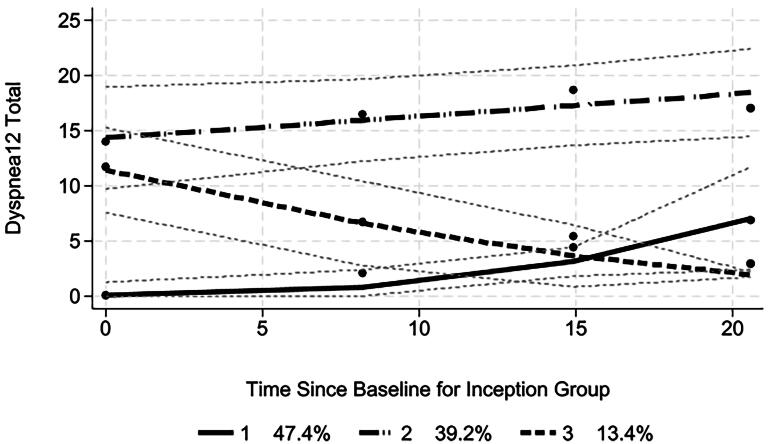
Dyspnea-12 trajectories in inception group (for whom duration since diagnosis ≤ 6 months). Fine dashed lines indicate 95% confidence limits.

The experience of breathlessness was also examined for those whose duration since diagnosis was six months or less—the “inception group” (Figure 3). Group 1 retained a low level of dyspnea over 20 months with a slight significant rise. Group 2 had a high level of dyspnea near diagnosis and showed a significant rise over time. However, group 3, with a similar intercept to group 2, showed a significant fall over time. This is associated with the observation that 48.7% of group 3 at first follow-up were in receipt of NIV, far higher than the other groups (*χ*^2^ 24.0 (df 2); *p* < 0.001). Almost all (98%) of this group also engaged with the follow-up compared to, for example, 27.4% of group 2 (*χ*^2^ 85.85 (df 2); *p* < 0.001).

### Minimal important change

The MIC/MCID was 4.5, which represented the median change of those who reported their breathing had become worse.

## Discussion

The Dyspnea-12 has been shown to have good fit to the Rasch model and provided all 12 items are answered, total, physical and affective interval level scores can be easily obtained from the transformation table. The availability of interval level scores is a key advantage of the Dyspnea-12 over other PROMs measuring dyspnea, which typically can provide only ordinal measurement. Change scores are critical in measuring the rate of progress of dyspnea or evaluating any benefit from treatment but change scores can only be calculated for interval level data.

Having both physical and affective components is important as different interventions may preferentially influence the physical or affective aspects of dyspnea. Recently, it has been shown that both physical and affective components of dyspnea were improved, at short and long term, by 8 weeks of individualized home-based pulmonary rehabilitation (36). The Dyspnea-12 scale has been adapted into many languages, albeit mostly with those who have cardiorespiratory disease (37–39). As there are many measures of dyspnea, it would be useful to see co-calibration of the different scales to obtain a common reference metric, so that the results from studies using different scales could be compared. One example has been co-calibration of Dyspnea-12 with DALS-15 (9). The MIC reported here is somewhat higher than other conditions such as COPD and asthma (40). This may be due to real difference among conditions or because those MIC were incorrectly calculated on ordinal data, which has been shown to introduce bias into MIC levels (35,41).

Analysis of our cohort of 1022 pwALS revealed three distinct groups following different trajectories of breathlessness, including one group who remained free from the symptom for the duration, and another with much higher levels which worsened over time. Those in this higher group were shown to have much higher symptomology, worse functioning, and poorer health status. Using the trajectory guided estimate of dyspnea, a prevalence of 12.6% (95% CI: 10.6–14.6) of severe breathlessness was derived from the Rasch-derived metric cut of 22.1 (ordinal cut 15).

The results of the present study were based on data collected in the United Kingdom but an international study in 15 countries showed considerable geographical variation in dyspnea from all conditions, even when adjusted for known risk factors and spirometry results, which only explained 13% of dyspnea variation (42). It would be useful to take this Dyspnea-12 metric cut point of 22.1 to see if prevalence was similar in other countries. TONiC-ALS data on dyspnea are currently being collected in USA, Australia and China.

There are many clinical implications of these findings, for patient monitoring and management. Clinical services must provide dyspnea monitoring which is customized to individual patient need; 57.3% of our cohort of pwALS had worsening dyspnea during 27 months follow-up and need regular, careful monitoring. Future work could assess whether the Dyspnea-12 may reduce the monitoring burden for pwALS. Timely access to interventions like NIV is crucial as provision was associated with reduction in dyspnea, as measured by Dyspnea-12. Conversely, some pwALS show stability and minor dyspnea over time so future work could explore whether remotely administering the Dyspnea-12 might safely reduce the frequency of respiratory testing.

Although we used a conservative criterion, of the upper quartile threshold of the deteriorating group, to define “severe” breathlessness (Dyspnea-12 metric score 22.1), only three-fifths of this severe group were receiving respiratory breathing support (King’s stage 4b). While one limitation of the study is that we do not know if these untreated participants with severe dyspnea were awaiting support, been offered but declined, or had trialed support and discontinued, any of these possibilities suggests different requirements for service development. An additional possibility is that the untreated participants with severe breathlessness did not meet prescribing criteria to receive respiratory support. In the 2016 National Institute for Health and Care Excellence guidelines for NIV use in motor neuron disease, respiratory assessment is recommended if pwALS have any symptoms of respiratory impairment and FVC <80% predicted value (43). While multicenter research shows a marked increase in dyspnea prevalence as FVC fell below 60% predicted, a proportion of people experience dyspnea when FVC ≥ 80% (42).

Strengths of this study are the large sample size, including the calibration sample of 1000 people for the Rasch analysis. This work provides transformation tables which allow users of the Dyspnea-12 to transform their ordinal raw scores to interval level estimates for parametric analyses. Furthermore, the association between dyspnea and King’s stage scoring adds to the evidence of the validity of the Dyspnea-12. Limitations include possible bias from the finding that those with higher levels of dyspnea were less likely to engage with the follow-up. Dyspnea may have been under-estimated if ameliorated by NIV by the next follow-up. Attrition, as expected, was high.

In conclusion, dyspnea is a cardinal symptom for pwALS, and can be quickly measured using the Dyspnea-12, whose results can easily be converted to interval level measurement. Both affective and physical aspects of breathlessness can be assessed. Dyspnea trajectories reveal different patterns, providing important information to improve patient care.

## Supplementary Material

Supplemental Material

## Data Availability

Data supporting this study are not openly available due to reasons of sensitivity and are available from the corresponding author upon reasonable request. Data are located in controlled access data storage at Walton Center NHS Trust. Please contact wcft.tonic@nhs.net.
