# Supplementary material for: Dyspnea (breathlessness) in amyotrophic lateral sclerosis/motor neuron disease: prevalence, progression, severity, and correlates
Source: Amyotroph Lateral Scler Frontotemporal Degener. 2024 Mar 11;25(5-6):475–85. doi: 10.1080/21678421.2024.2322545 (PMC11286210; doi:10.1080/21678421.2024.2322545)
Supplement: Supplemental Material [file IAFD_A_2322545_SM6491.pdf]

Supplementary Materials

This supplementary material is from:

Dyspnea (breathlessness) in Amyotrophic Lateral Sclerosis/Motor Neuron Disease: Prevalence, progression, severity and correlates

CA Young, A Chaouch, CJ McDermott, A Al-Chalabi, SK Chettri, K Talbot, T Harrower, R Orrell, J Annadale, CO Hanemann, A Scalfari, A Tennant, RJ Mills,
On behalf of the TONiC-ALS study group

Amyotrophic Lateral Sclerosis and Frontotemporal Degeneration 2024;
doi.org 10.1080/21678421.2024.2322545

Contents

1.1. Methods of Rasch Analysis 1
1.2. Methods of Trajectory Analysis 3
Supplementary File: Table S1. Strategies seeking fit of the data to the Rasch model. 4
1.3. References 4

1.1. Methods of Rasch Analysis

Data from each (sub)scale was tested against the requirements of the Rasch Measurement model [1]. Briefly, these requirements include i) unidimensionality; ii) monotonicity; iii) homogeneity; iv) local independence and v) group invariance [2, 3]. Whichever set of items are to be added together to provide a score, they should satisfy all these requirements. They should: i) measure one thing (domain/construct/trait; ii) the probability of a positive response to an item (or in the case of polytomous items, the transition from one response category to the next) should increase with underlying ability, as should the total score [4]; iii) the same hierarchical ordering of items should hold for each level (or grouping) of the score [5]; iv) items should be conditionally (on the score) independent of one another [6]; and v) the response to items across different groups such as age or gender should, conditioned on the total score, be the same – referred to as (the absence of) Differential Item Functioning (DIF) [3].

Each requirement is tested. A t-test is used to determine if two separate groups of items deliver significantly different estimates, following the procedure given by Smith [7].

The hierarchical ordering of items across the scale is determined through a Chi-Square test of fit based on grouped scores. Monotonicity is evaluated through inspection of the item-category ordering. Conditional item dependence is determined though the correlation of residuals, where pair-wise correlations should not exceed 0.2 above the average residual [8]. Should clusters of locally dependent items be found, consideration is given to grouping these into 'super items' or testlets (simply adding them together to make one larger item, the latter based on *a priori* defined groups) to absorb the local dependency [9]. In the RUMM2030 software, this gives a bi-factor equivalent solution retaining a specified proportion of the variance. This "Explained Common Variance (ECV)" is reported, whereby a value less than 0.7 is indicative of requiring a multidimensional model, a value ≥0.9 a unidimensional model, and the grey area in between, undetermined, requiring further evidence [10]. Consequently, value of the ECV at 0.9 and above is considered acceptable in the current analysis. If two parallel forms are created from either a subscale structure, if present, or from the pattern of local dependency in the item set, this requires a latent correlation ≥ 0.9. This is consistent with the reliability required for individual use [11]. Consequently, valid parallel forms would require both their latent correlation to be ≥0.9 and the ECV to be ≥0.9.

Group invariance (DIF) is tested through an ANOVA of residuals for age, gender, duration since diagnosis, education levels, and whether the patient is self-employed or employed, and working full-time or part-time. Should DIF be identified, it is tested by a comparison of person estimates from split and unsplit solutions to see if it is 'substantive' [12]. Where the difference is significant (a paired t-test), the result is reported as an effect size where a value higher than 0.1 is considered to represent substantive DIF [13]. If this is present, then the scale works in different ways for the contextual factor under consideration, and results are reported separately. Finally, reliability is reported as both a Person Separation Index (PSI), and as Cronbach's alpha. If data are normally distributed they are equivalent, but otherwise PSI tends to be lower the more data are skewed.

Values are treated the same, and so values below 0.7 would be described as low, as they do not support group use.

A hierarchical approach to seeking fit of the data to the model for existing scales is adopted, with level 1 as the priority (Supplementary file:Table 1). All aspects listed above must be met. Should a level 5 solution be unavailable, item deletion will be considered (level 6). If this fails then level 7 will be utilised to test if the scale satisfies ordinal scaling; if not level 8 is remains, indicating failure.

1.2. Methods of Trajectory Analysis

A group-based trajectory model was applied, which is designed to identify groups of individuals following similar developmental trajectories [14, 15]). It was implemented through traj.ado in STATA17 [16]. The number and shape (via polynomial functions) of trajectories were determined by analysing one to five group models without covariates. To accommodate attrition, a 'dropout' model was applied, specified in its basic form of variable dropout across assessment occasions [17]. The Bayesian Information Criterion (BIC) was used to determine the best-fitting model, also with consideration for a useful and parsimonious model. Average posterior probabilities above 0.7 were also deemed to indicate optimal fit [18]. Missing data were handled using a maximum likelihood approach based on a missing-at-random assumption.

Supplementary File: Table S1. Strategies seeking fit of the data to the Rasch model.

Level	Nature	Adjustments	Reporting		
			Chi-Square	ECV ≥0.9	Latent Correlation ≥0.9
1	Item-based	None	Interaction	No	No
2	Item-Based	Clusters for Local Item Dependency	Interaction	Yes	No
3	Domain-based	On existing sub-scales >2	Interaction	Yes	No
4	Parallel Form	On existing sub-scales ≤2, or 2 local dependency patterns or conceptual groups	Conditional	Yes	Yes
5	Parallel Form	On alternative items	Conditional	Yes	Yes
6	Item Deletion	On reduced items, and repeating Levels 1-5	Interaction	No	No
7	Mokken Scaling	On items if Unidimensional. Loevinger's coefficient H ≥0.4-moderate	No	No	No
8	Fail	No valid ordinal scale	No	No	No

1.3. References

1. Rasch G. Probabilistic Models for Some Intelligence and Attainment Tests. Chicago: The University of Chicago Press; 1980.
2. Gustafsson J. Testing and obtaining fit of data to the Rasch model. British Journal of Mathematical & Statistical Psychology. 1980;33(2):205-33.
3. Teresi JA, Kleinman M, Ocepek-Welikson K. Modern psychometric methods for detection of differential item functioning: application to cognitive assessment measures. Stat Med. 2000;19(11-12):1651-83.
4. Kang HA, Su YH, Chang HH. A note on monotonicity of item response functions for ordered polytomous item response theory models. The British journal of mathematical and statistical psychology. 2018;71(3):523-35.

5. Rost J. An unconditional likelihood ratio for testing item homogeneity in the Rasch model. Education Research and Perspectives. 1982;9(June):7-17.
6. Wilson M. Detecting and Interpreting Local Item Dependence Using a Family of Rasch Models. Applied psychological measurement. 1988;12(4):353-64.
7. Smith E. Detecting and evaluating the impact of multidimensionality using item fit statistics and principal component analysis of residuals. J Appl Meas. 2002;3:205-31.
8. Christensen KB, Makransky G, Horton M. Critical values for Yen's Q3: Identification of local dependence in the Rasch model using residual correlations. Applied psychological measurement. 2017;41(3):178-94.
9. Wainer H, Kiely G. Item clusters and computer adaptive testing: A case for testlets. J Educ Meas. 1987;24(3):185-202.
10. Quinn H. Bifactor Models, Explained Common Variance (ECV), and the Usefulness of Scores from Unidimensional Item Response Theory Analyses [Masters Thesis]. North Carolina: University of North Carolina at Chapel Hill; 2014.
11. Bland JM, Altman DG. Statistics notes: Cronbach's alpha. British Medical Journal. 1997;314:572.
12. Hagquist C, Andrich D. Recent advances in analysis of differential item functioning in health research using the Rasch model. Health Qual Life Outcomes. 2017;15(1):181.
13. Rouquette A, Hardouin JB, Vanhaesebrouck A, Sébille V, Coste J. Differential Item Functioning (DIF) in composite health measurement scale: Recommendations for characterizing DIF with meaningful consequences within the Rasch model framework. PLoS One. 2019;14(4):e0215073.
14. Jones BL, Nagin DS. A note on a Stata plugin for estimating group-based trajectory models. Sociological Methods & Research. 2013;42(4):608-13.
15. Mori M, Krumholz HM, Allore HG. Using latent class analysis to identify hidden clinical phenotypes. JAMA. 2020;324(7):700-1.
16. StataCorp. Stata Statistical Software. Release 15 ed. College Station, TX: StataCorp LLC; 2017.
17. Haviland AM, Jones BL, Nagin DS. Group-based trajectory modeling extended to account for nonrandom participant attrition. Sociological Methods & Research. 2011;40(2):367-90.
18. Nagin DS, Odgers CL. Group-based trajectory modeling in clinical research. Annual review of clinical psychology. 2010;6:109-38.
